# Analysis of the impact of solvent on contacts prediction in proteins

**DOI:** 10.1186/1472-6807-9-22

**Published:** 2009-04-15

**Authors:** Sergey A Samsonov, Joan Teyra, Gerd Anders, M Teresa Pisabarro

**Affiliations:** 1 Structural Bioinformatics, BIOTEC TU Dresden, Tatzberg 47-51, 01307 Dresden, Germany

## Abstract

**Background:**

The correlated mutations concept is based on the assumption that interacting protein residues coevolve, so that a mutation in one of the interacting counterparts is compensated by a mutation in the other. Approaches based on this concept have been widely used for protein contacts prediction since the 90s. Previously, we have shown that water-mediated interactions play an important role in protein interfaces. We have observed that current "dry" correlated mutations approaches might not properly predict certain interactions in protein interfaces due to the fact that they are water-mediated.

**Results:**

The goal of this study has been to analyze the impact of including solvent into the concept of correlated mutations. For this purpose we use linear combinations of the predictions obtained by the application of two different similarity matrices: a standard "dry" similarity matrix (DRY) and a "wet" similarity matrix (WET) derived from all water-mediated protein interfacial interactions in the PDB. We analyze two datasets containing 50 domains and 10 domain pairs from PFAM and compare the results obtained by using a combination of both matrices. We find that for both intra- and interdomain contacts predictions the introduction of a combination of a "wet" and a "dry" similarity matrix improves the predictions in comparison to the "dry" one alone.

**Conclusion:**

Our analysis, despite the complexity of its possible general applicability, opens up that the consideration of water may have an impact on the improvement of the contact predictions obtained by correlated mutations approaches.

## Background

The correlated mutations concept was introduced in the 90s [1-4] and has been widely used for protein contacts prediction [5]. The method is based on the assumption that interacting protein residues co-evolve, so that a mutation in one of the interacting counterparts is compensated by a mutation in the other. Therefore, it is possible to introduce an exchange matrix or other measures of similarity for each sequence position in a multiple sequence alignment and to use covariance (correlation coefficient) between two positions to predict if the residues at these positions may establish physical contact in 3D space, and develop contact maps. Several different similarity measures and algorithms have been implemented in the concept of correlated mutations [5-7]. Most exchange matrices are based either on physico-chemical properties of amino acids or on statistical data on the substitutions obtained from multiple sequence alignments [8]. Statistically it is clear that the distribution of distances between the residues at highly correlated positions is shifted towards lower values compared to the distance distribution of all residues. This has been demonstrated in the study of correlated mutations for residues within one protein domain (intradomain), for residues from different domains in multidomain proteins (interdomain intraprotein) [9,10] and in transmembrane proteins [11]. At the same time, attempts to use the concept of correlated mutations to predict thermodynamically coupled residues have suggested that the method is successful only for residues in evolutionary constrained positions [12].

The concept of correlated mutations has been intensively developed recently. The implementation of neural nets into algorithms of contact predictions has allowed to substantially improve the accuracy of the methods in a number of studies [13-16]. Also the application of filtering procedures such as the similarity of sequences in a dataset and the number of sequences in multiple sequence alignments, introduction of weights for physico-chemical properties of the residue pairs and creation of sub-multiple sequence alignments were successfully used to increase a true positive ratio of contact predictions [17]. Nowadays, different correlated mutations based approaches yield predictions accuracies in the range of 0.1–0.4 [17] but they are still of little use in the *ab initio *prediction of protein structure [7].

Previously, we have shown that water-mediated interactions play an important role in protein interfaces [18,19]. In particular, we observed that the interfacial residues interacting only through one water molecule (wet spots) are more similar in terms of dynamic and energetic properties to residues in the core of proteins than to residues on the protein surface. Moreover, in our studies interfacial water molecules show significantly longer residence times than water molecules on the protein surface or in bulk solvent, and have been shown to give an indispensable energetic impact on complex formation [19]. In other studies it has been demonstrated that inclusion of solvent term into the Hamiltonian of protein systems has improved folding predictions compared to *in vacuo *folding models [20]. Also consideration of solvent explicitly in protein docking approaches has recently shown promising results [21]. In addition, we have observed that water molecules in protein interfaces may contribute to the conservation of interactions by allowing more sequence variability in the interacting partners. In particular, we have observed water-mediated interactions in protein complex interfaces that are not predicted by "dry" correlated mutations approaches [19]. Interestingly, in one of the recent studies on correlated mutations, protein contacts prediction has been shown to be more accurate for protein cores than for the whole protein [22]. This could be partly explained by a higher conservation of residue contacts in protein cores, especially the hydrophobic ones [23] and probably also by the fact that the participation of solvent in protein contacts is being ignored.

The goal of this study has been to analyze the impact of including solvent into the concept of correlated mutations. For this purpose, we use a linear combination of predictions obtained by the use of two similarity matrices: a standard and widely used "dry" similarity matrix (DRY) [24] and a "wet" similarity matrix (WET) derived from data on all water-mediated protein-protein interfacial interactions in the PDB [25]. We compare the predictive results obtained with different combinations of these two similarity matrices in terms of number of correctly predicted contacts, accuracy, improvement ratio over random prediction for intradomain contacts and distributions of distances between residues in interdomain pairs.

Our results show that, despite a partial interdependence of both WET and DRY matrices, there is a clear trend pointing that a combination of these two matrices yields improved predictions over the single use of the DRY matrix for both intra- and interdomain contacts. The results obtained in this work underline the importance of water-mediated interactions in the description of protein-protein interactions, and that implementing combinations of "dry" and "wet" matrices could possibly improve the results obtained by correlated mutations-based approaches.

## Results and discussion

### Residue-solvent relations in proteins

**Figure 1:**
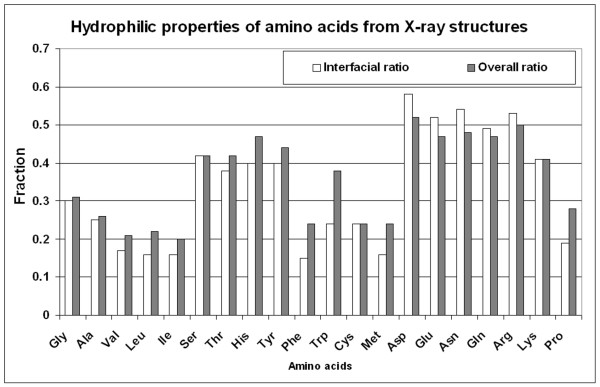
**Water contacts of residues in PDB**. Fractions of residues found to be in contact with water in protein interfaces (white) and in whole proteins (grey) in the PDB.

Independently of residue types, we calculated the average ratios between the number of residues found to be in contact with water and all residues in X-ray PDB structures. A negligible difference was found between these ratios for interfaces and the whole protein (0.33 and 0.35, respectively). The ratios by residue type (Figure 1 and see additional file 1) correlate with an adjusted squared correlation coefficient R^2 ^= 0.90 (p-value~10^-10^) and there is also a clear trend of residue ratios distribution in interfaces, which relates to their hydrophilic properties. This agrees with observations obtained from other datasets not including the whole PDB [26]. The better correlation between the ratios and the hydrophilicity index for interfaces compared to the whole protein (R^2 ^= 0.62 p-value~10^-5 ^and R^2 ^= 0.44 p-value~10^-3^, respectively) could be explained by the fact that the whole protein includes many residues in the core that are not accessible to water. This further supports the evidence that residue-solvent relations in protein interfaces are different from the ones in the proteins as a whole [18,19].

### Relations between the DRY and WET similarity matrices

Both DRY and WET similarity matrices are created in a way that each column or row is a vector, which coordinates correspond to the similarity between certain amino acid residue type and other residue types. It is possible to define whether these vectors are interdependent for both matrices by application of linear regression analysis. The data obtained and averaged for all types of residues are presented in Table 1. High degree of correlation is observed for some vectors, which correspond to hydrophilic residues (excluding Thr and Tyr) and for Ile, Leu, Met, Val, suggesting that these vectors in the matrices are close to be collinear in 20-dimensional space. This can be explained by the properties of these residues. In particular, hydrophilic residues interact by electrostatic forces through their polar atoms, and water mediation in this case can only change the electrostatic forces by introducing water dipoles oriented in a way to weaken the initial electric field. For hydrophilic residues there is a correlation between hydrophilicity indexes and co-linearity of the corresponding vectors in the DRY and WET matrices, which explains also relatively low co-linearity for Tyr and Thr residues in comparison to other hydrophilic residues (additional file 2). Direct and water-mediated interactions formed by main chains of Ile, Leu, Met and Val in interfaces have been previously shown to be especially important, whereas other residues that present no correlation have been shown to predominantly participate in side-chain interactions in interfaces [18]. We conclude that the DRY and WET similarity matrices contain partially interdependent information for some of amino acid residues, and the found similarities can be explained by the physico-chemical properties of these residues.

**Table 1:** Correlation between vectors per residue type in the DRY and WET matrices.

Residue	p-value	Adjusted R^2^
Ala	0.90	-0.05

Arg	4·10^-3^	0.35

Asn	4·10^-5^	0.65

Asp	6·10^-4^	0.46

Cys	0.14	0.07

Gln	5·10^-4^	0.47

Glu	4·10^-4^	0.49

Gly	0.53	-0.03

His	0.02	0.22

Ile	8·10^-4^	0.44

Leu	6·10^-3^	0.31

Lys	8·10^-3^	0.29

Met	6·10^-3^	0.31

Phe	0.02	0.24

Pro	0.62	-0.04

Ser	2·10^-3^	0.39

Thr	0.07	0.12

Trp	0.18	0.05

Tyr	0.71	-0.05

Val	4·10^-3^	0.33

### Intradomain contacts prediction

Our dataset for intradomain contacts prediction consisted of domains of 50 PFAM protein families (Table 2). The lengths of the reference sequences varied from 30 to 195 residues. Initially we analyzed L, L/2, L/3, L/5 and L/10 best correlated contacts for each family (L is the length of the reference sequence). The number of sequences considered for the multiple sequence alignments was in the range of 20 to 295 sequences. Previous studies have shown that accuracy (ratio between the number of correctly predicted contacts and the number of total predicted contacts) and improvement ratio over random prediction (ratio between accuracy and the probability of predicting a contact by chance) decrease with the increase of the number of analyzed contacts [4-6]. Table 3 shows accuracy and improvement ratio over random prediction for α = 0.5 (weight for WET matrix prediction when for DRY is 1), which corresponds to the average best accuracy obtained for different numbers of analyzed predicted contacts. The results obtained for other α values followed the same trend (data not shown). Independent of the number of analyzed contacts the best predictions in average did not correspond to α = 0. The obtained values for accuracy and improvement ratio over random prediction are within the ranges obtained by other correlated mutations approaches [17,22]. However, direct quantitative comparison of these methods is not appropriate because of their substantial differences in their residue contacts definitions. In particular, some of these approaches utilize for contact definition (see contact definition in Methods section) a chosen distance cut-off of 6–8 Å between atoms [4,16,17], whereas we use physico-chemical properties of protein residues, which results in a ≤ 4 Å cut-off [27].

**Table 2:** Dataset used for intradomain contact predictions.

PFAM ID	PDB ID^a^	R (Å)	N^b^	% id^c^	L^d^	Ran acc^e^	Acc^f^	R^g^	Opt α^h^	X_d dry_^i^	Opt_Xd _α^j^	X_d wet_|_opt α_^k^
PF00014	6PTI	1.70	151	33	52	0.096	0.346	3.61	1	9.37	1	11.16

PF03705	1AF7	2.00	85	20	57	0.081	0.241	2.65	0.5, 4, 10	6.14	2	7.63

PF00062	5LYZ	2.00	22	46	127	0.043	0.078	1.91	0, 0.5	2.68	0	2.68

PF00018	1BU1	2.60	61	28	56	0.088	0.357	4.06	0.5	12.99	0	12.99

PF03900	1PDA	1.76	21	25	74	0.062	0.237	3.82	2	9.18	0.2	9.99

PF00034	1CTJ	1.10	35	17	89	0.061	0.250	4.10	1	9.13	0.1	10.34

PF01568	1DMR	1.82	88	18	113	0.044	0.050	1.14	0.2, 0.5	10.62	2	12.53

PF00127	8PAZ	1.60	31	29	89	0.055	0.102	1.85	2	0.50	1	4.82

PF01814	2MHR	1.30	295	12	49	0.098	0.400	4.08	0.5, 2	8.39	2	13.14

PF00017	1BMB	1.80	59	28	93	0.058	0.212	3.66	0 – 0.5	5.98	1	8.37

PF01320	1AYI	2.00	45	47	86	0.056	0.233	4.15	0.2	16.04	0	16.04

PF08666	1AME	1.65	171	14	66	0.074	0.273	3.69	0	10.25	0	10.25

PF01337	1A19	2.76	30	25	89	0.065	0.178	2.87	0, 0.1	4.55	0.1	4.72

PF00595	2HB2	2.30	56	19	85	0.062	0.233	3.75	0.5 – 2	10.16	1	11.67

PF00531	1WMG	2.10	92	14	82	0.066	0.250	3.79	0 – 0.5	7.67	0.2	7.95

PF00397	1EG3	2.00	73	32	30	0.143	0.467	3.26	2 – 20	6.59	2	8.81

PF01335	2F1S	1.40	40	21	76	0.072	0.237	3.88	0.1, 0.2	5.66	0.2	5.96

PF00619	1CY5	1.30	61	16	85	0.066	0.209	3.43	0.2 – 2	5.09	2	9.42

PF02213	1SYX	2.35	112	28	58	0.083	0.241	2.91	0.5 – 2	7.37	0.5	7.77

PF05743	1UZX	1.85	28	27	118	0.035	0.068	1.98	0.1	7.22	0	7.22

PF00536	1B4F	1.95	69	28	74	0.076	0.395	5.19	0.2 – 2	15.53	2	16.36

PF03114	1ZWW	2.30	29	19	195	0.021	0.074	3.53	0.2	2.41	20	3.99

PF00169	1NTY	1.70	139	10	112	0.050	0.071	1.43	0, 0.2, 0.5	5.46	2	7.53

PF08416	1WVH	1.50	49	28	132	0.040	0.106	2.65	2, 4	0.53	0.1	1.24

PF01981	1WN2	1.20	69	43	116	0.049	0.172	3.52	0.1 – 0.5	7.63	20	12.38

PF03992	1XBW	1.90	116	15	65	0.068	0.125	1.84	0.5	3.34	0	3.34

PF00907	1H6F	1.70	23	49	183	0.032	0.033	1.03	0 – 20	3.30	2	6.03

PF02237	1WPY	1.60	47	21	48	0.094	0.167	1.77	0.5 – 2	-2.83	0.5	0.22

PF08031	2AXR	1.98	64	34	34	0.135	0.235	1.74	0.1, 0.2	-0.05	2	3.37

PF02861	1K6K	1.80	165	21	51	0.098	0.440	4.49	1, 4, 10, 20	9.55	20	13.21

PF02834	1VGJ	1.94	106	14	85	0.048	0.119	2.48	4 – 20	-0.51	4, 10	3.21

PF01423	1KQ1	1.55	128	23	60	0.079	0.167	2.11	0.2, 0.5	5.78	0.1, 0.2	7.14

PF01472	1AS0	1.80	106	24	78	0.058	0.128	2.21	1 – 20	3.57	2, 4	11.45

PF01909	1NO5	1.80	119	14	91	0.059	0.133	2.26	0.1 – 1	4.97	0.2	6.01

PF09261	1R34	1.95	79	31	78	0.069	0.205	2.97	0.1, 0.2	4.87	0.1	6.64

PF01315	1VLB	1.28	28	19	117	0.041	0.207	5.05	1, 2	7.70	2	10.28

PF04545	1KU3	1.80	128	31	54	0.096	0.370	3.86	0, 0.1, 1, 10, 20	12.37	10, 20	12.76

PF00984	1MV8	1.55	24	17	98	0.048	0.184	3.83	0.5 – 20	8.27	0.2	9.78

PF01658	1U1I	1.90	20	31	105	0.049	0.096	1.96	0.1 – 20	1.93	0.5	6.28

PF00745	1GPJ	1.95	34	23	99	0.048	0.100	2.08	0.1 – 0.5	3.17	0.1	4.17

PF03099	1WNL	1.60	65	14	117	0.043	0.121	2.81	0	13.7	0.2	14.20

PF01985	1JO0	1.37	50	23	84	0.064	0.167	2.60	0 – 0.2	6.96	0	6.96

PF08436	1Q0Q	1.90	77	57	94	0.049	0.213	4.34	0 – 0.1	6.91	10	10.15

PF02881	1JPN	1.90	52	19	85	0.063	0.119	1.89	0 – 20	3.94	2	5.78

PF01966	1YNB	1.76	158	12	91	0.057	0.333	5.85	0 – 0.2	-0.79	2	2.20

PF00191	1YII	1.42	178	28	66	0.076	0.273	3.59	0 – 0.2	-0.35	10	1.05

PF00317	1XJE	1.90	79	23	90	0.056	0.178	3.17	0.5 – 2	10.01	0.5	13.16

PF00046	1PUF	1.90	184	37	60	0.082	0.333	4.07	1, 2	6.07	2	8.60

PF00077	5FIV	1.90	48	27	108	0.049	0.093	1.89	2	-1.37	1	3.63

PF00042	1ECN	1.40	73	18	101	0.046	0.163	3.56	1, 2	6.89	2	7.19

^a^PDB ID; ^b^Number of sequences; ^c^Average sequences pairwise similarity (%); ^d^Reference sequence length; ^e^Random accuracy; ^f^Accuracy for optimal *α*; ^g^Improvement ratio over random prediction for optimal *α*; ^h^Values for *α *= 0; ^i^*α *corresponding to the highest accuracy; ^j^*α *corresponding to the highest X_d_; ^k^X_d _highest value.

**Table 3:** Prediction parameters dependence on the number of analyzed contacts.

Predicted contacts analyzed	Accuracy	Improvement ratio over random prediction
L	0.15 ± 0.09	2.24 ± 0.95

L/2	0.18 ± 0.10	2.67 ± 1.08

L/3	0.19 ± 0.12	2.81 ± 1.52

L/5	0.21 ± 0.16	3.16 ± 1.79

L/10	0.23 ± 0.20	3.55 ± 2.81

L is the length of the reference sequence. The value *α *= 0.5 has been used.

**Figure 2:**
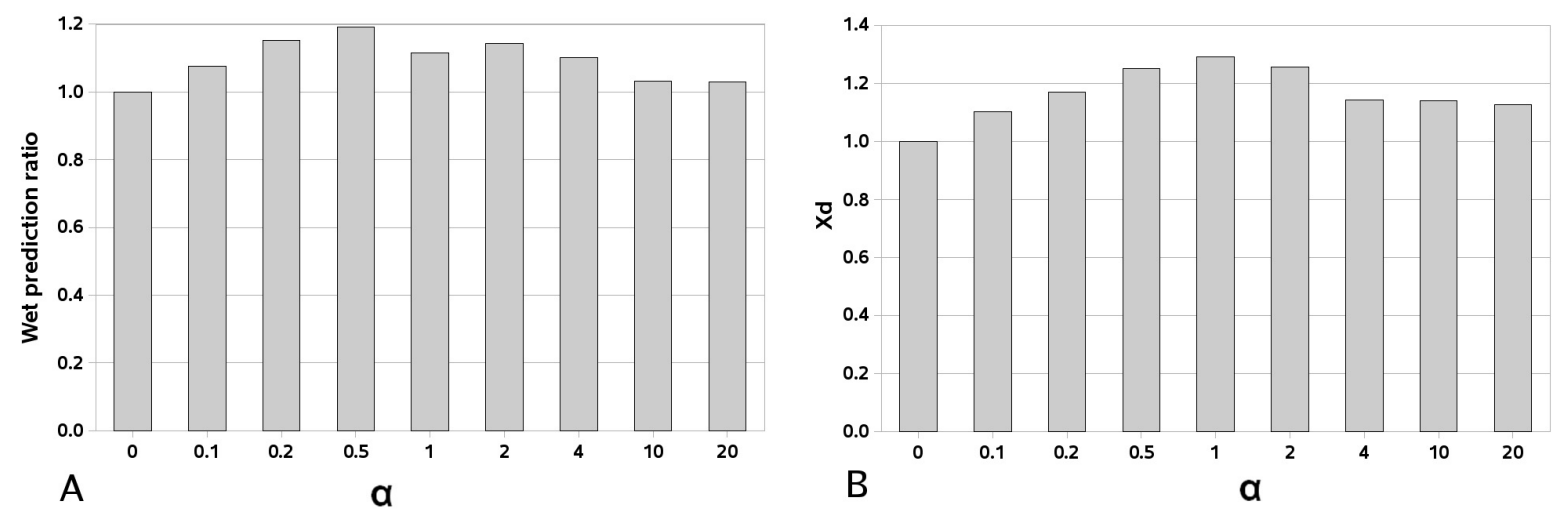
**Dependence on *α *of relative prediction characteristics for the intradomain dataset**. A) Wet prediction ratio. B) Relative harmonic weighted difference statistic (*X*_*d*_).

We compared the dependences on *α *of: i) accuracy, ii) improvement ratio over random prediction, iii) number of correctly predicted contacts (C_corr_); and, since our dataset is heterogeneous (see high standard deviations in Table 3), we normalized these parameters by the corresponding values at *α *= 0 (wet prediction ratio). For the purpose of wet prediction ratio comparison at different values of *α *we found L/2 to be the most appropriate number of contacts. This choice is explained by the fact that the changes in prediction results influenced by α variation become hardly detectable if a smaller number of contacts (C_total_) is considered for analysis since these changes are limited by low values of C_total _and, consequently, of correctly predicted contacts (C_corr_). On the other hand, the increase of C_total _generally leads to decrease of prediction accuracy and to negligible differences in prediction results corresponding to different *α *values. Only in 2 out of the 50 families of our dataset best predictions correspond to *α *= 0 values (Table 2). Maximum values for wet prediction ratio and relative *X*_*d *_(harmonic weighted difference statistic) averaged for the whole dataset are obtained when *α *= 0.5 and *α *= 1 (1.19 and 1.29, respectively; Figure 2A, B). This means that, for these values of *α*, introduction of the WET similarity matrix improves prediction by 20–30% on average. Noticeably, the high values of *α *∈ {10, 20} still make the predictions on average better than by the single use of the DRY matrix. For optimal value *α *= 0.5, absolute values of accuracy and improvement ratio over random prediction averaged for all 50 families increase by 1.4% and 0.19, respectively, in comparison to the single use of the DRY similarity matrix. For each family in the dataset there is an essentially higher increase of accuracy and improvement ratio over random prediction than on average. In some families, wet prediction ratio is improved more than twice (reference structures 1AF7, 1PDA, 8PAZ, 1DMR, 1AS0) and even 4.5 times (reference structure 1WVH) when *α *> 0. Our results show a significant improvement (20–30% of increase in wet prediction ratio) in predictions by the introduction of the WET similarity matrix in comparison to the single use of the DRY matrix within a correlated mutations approach. We observe that for sequence separations |i-j| > 6, 12, 24 our results follow the same trend. The obtained results for *α *= 0.5 for different number of contacts (L, L/2, L/3, L/5, L/10) are shown in Table 4. We observe that the best predictions correspond to *α *= 0.2 and 0.5 for most of sequence separation values and number of contacts. Wet prediction ratios for the whole range of analyzed *α *are presented in a figure in supplementary material (additional file 3). In all cases, independently of sequence separation and number of contacts, the best predictions correspond to *α *> 0.

**Table 4:** Accuracy, improvement ratio over random prediction and wet prediction ratio for different sequence separations.

	Sequence separation 6			Sequence separation 12			Sequence separation 24		
	Accuracy	R	Wet ratio	Accuracy	R	Wet ratio	Accuracy	R	Wet ratio

L	0.061	3.07	1.01	0.051	3.02	1.02	0.042	2.97	1.06

L/2	0.079	4.18	1.11	0.070	4.34	1.14	0.050	3.76	1.10

L/3	0.087	4.56	1.14	0.071	4.49	1.01	0.060	4.61	1.14

L/5	0.099	5.49	1.05	0.085	5.71	1.08	0.068	5.18	1.04

L/10	0.122	6.68	1.14	0.103	6.89	1.13	0.078	6.31	1.00

L is the length of the reference sequence. R is improvement over random prediction. The value *α *= 0.5 has been used.

### Interdomain contacts prediction

**Figure 3:**
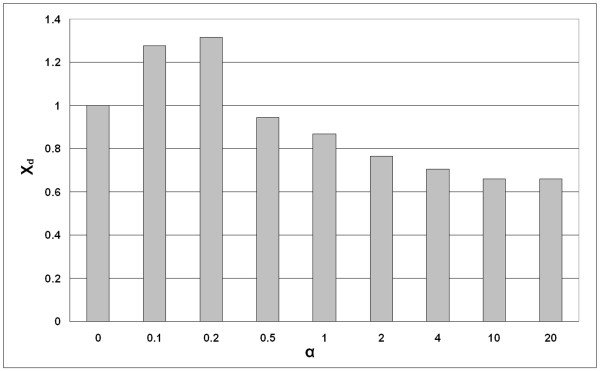
**Predictions for interdomain dataset**. Relative harmonic weighted difference statistic (*X*_*d*_) dependence on *α*.

**Figure 4:**
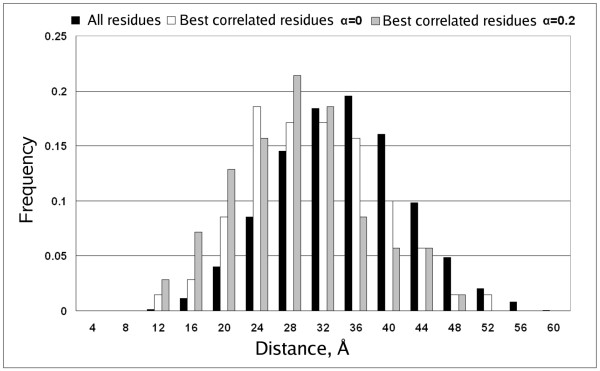
**Proportion of residue pairs at distance bins for the interaction SH2-SH3**. All residue pairs are shown in black, correlated pairs with *α *= 0 in white, and correlated pairs with *α *= 0.2 in grey. Reference structure used is PDB ID 2SRC.

The interdomain dataset used for our studies consisted of 10 different pairs of interacting domains (Table 5). From the analysis of the (L_1_+L_2_)/2 predicted interdomain residue contacts (L_1 _and L_2 _are the lengths of the sequences in each of the two domains) we observed that in 9 out of 10 cases best predictions in terms of *X*_*d *_were obtained when both the WET and DRY matrices were used. Relative *X*_*d *_averaged for the whole dataset reaches a maximum value of 1.32 at *α *= 0.2 and then decreases with the further increase of *α *(Figure 3). In one of the examples (SH2-SH3 domains interaction) the differences of distance distributions for different α values are dramatic (Figure 4). In this case the *X*_*d *_value for predicted contacts at *α *= 0 and *α *= 0.2 changes almost twice (Table 5). These results point out that the use of the WET similarity matrix might improve the statistic *X*_*d *_in comparison to the single use of the DRY similarity matrix.

**Table 5:** Dataset used for interdomain contact predictions.

Interacting partners	PFAM ID1/ID2	PDB ID^a^	N^b^	% iden^c^	L_1_^d^	L_2_^e^	X_d dry_^f^	Opt_Xd _α^g^	X_d wet_|_opt α_^h^
Tyrosine kinase SH3/SH2 domains	PF00018/PF00017	2SRC	19	35	57	83	1.86	0.2	3.25

Alcohol dehydrogenase N-/C-domains	PF08240/PF00107	1ADG	89	23	128	143	3.52	0.2	3.64

Mg superoxide dismutase N-/C-domains	PF00081/PF02777	1AP5	23	44	82	107	4.76	0.2	5.04

Immunoglobulin heavy/light chains	PF00047/PF00047	12E8	116	36	107	114	13.56	0	13.56

Ortnithine transferase N-/C-domains	PF02729/PF00185	1DUV	20	30	142	178	4.47	0.1	4.94

NFKB factor RHD/TIG domains	PF00554/PF01833	1SVC	21	40	199	100	4.56	0.5	4.62

STAT alpha/binding domains	PF01017/PF02864	1BF5	32	38	180	251	4.30	0.2	4.42

Mur-ligase catalytic/C-terminal domains	PF01225/PF08245	1E8C	26	25	82	208	1.84	0.1	2.12

Dynamin central/N-domains	PF00350/PF01031	2AKA	32	40	174	89	0.04	0.2	0.14

Trk C-/N-domains	PF02254/PF02080	1LNQ	42	20	114	72	0.53	1	0.78

^a^PDB ID of the reference structure; ^b^Number of sequences in the multiple sequence alignment; ^c^Average percentage of sequences pairwise similarity; ^d, e^Lengths of the reference sequences; ^f^Values for *α *= 0; ^g^*α *value corresponding to the highest X_d_; ^h^X_d _highest value.

Dependence of relative average *X*_*d *_on *α *for interdomain contacts prediction (Figure 3) resembles the one obtained for intradomain prediction (Figure 2B) but they differ in the optimal *α *and in the *X*_*d *_corresponding to the higher *α *values. While in predictions of intradomain contacts all values of *α *> 0 lead to the improvement of contact predictions, in the case of interdomain contacts prediction the use of the WET similarity matrix yields higher *X*_*d *_than the DRY alone when *α *∈ {0.1,0.2}. This might be due to the differences in distance distributions between the analyzed pairs of residues, which are closer to each other in the case of intradomain contacts. Nevertheless, introduction of the WET similarity matrix improves contact prediction compared to the single use of the DRY similarity matrix for both intra- and interdomain contacts. Although there are still significant limitations for practical use of the correlated mutations approach for interdomain contacts prediction, also mentioned by other authors [5,9], we believe that consideration of water by the use of "wet" similarity matrices could improve the results obtained by correlated mutations approaches.

## Conclusion

This study is the first investigating the impact of inclusion of solvent into the concept of correlated mutations. With this work we further demonstrate our previous observations that relations between solvent and protein residues in protein interfaces differ from those in the whole protein. Recent work on bond preferences in inter- *versus *intraprotein interactions highlights the different architecture of protein interfaces and their unique bond preferences [28].

Two similarity matrices have been used in this work: the McLachlan matrix as the DRY similarity matrix and a WET similarity matrix derived by statistical analysis of the frequency of water contacts by residue type in protein interfaces in the whole PDB. Analysis of the DRY and WET similarity matrices shows that they are interdependent for some residue types, which could be explained by physico-chemical properties of individual amino acid residues. We analyze two datasets containing 50 domains and 10 domain pairs belonging to PFAM families. We sum the predictions obtained by the use of both matrices with different weight coefficients and find optimal combinations for best predictions. Our datasets are heterogeneous to propose one best weight value to be able to apply the optimized method to all domain families; however, the prediction of contacts obtained by the introduction of the WET similarity matrix is improved for most of the families in the datasets (for both intra- and interdomain) as well as on average (by 20–30%). Our analysis of solvent impact on contact prediction in proteins suggests that further development of the correlated mutations concept would benefit from taking into account solvent as an active participant in protein-protein interactions, which is usually overlooked in these studies.

## Methods

### Dataset and multiple sequence alignments

We based the generation of our dataset on previous similar studies [4,9,22]. Our dataset includes 50 domains and 10 domain pairs extracted from the PFAM database [29]. Consecutive increase of the size of our dataset for intradomain contacts did not significantly change our results.

For most of the families, only seed sequences were used, except for the cases when the number of seed sequences was less than 20. Datasets with a smaller number of sequences are not supposed to be useful in correlated mutations analysis [22]. The *reference sequence *(corresponding to the structure used for predictions evaluation) was added to the set of sequences, if this did not already contain it, following the same procedure that Eyal and co-workers used for obtaining a substitution matrix for protein structure prediction purposes [22]. Multiple sequence alignments were obtained with CLUSTALW [30]. Sequences with more than 95% of identity were not taken into account.

For the interdomain dataset the sequences from the two domain families were aligned independently. Except for the case of immunoglobulins, where light and heavy chains were used as two interacting domains, all interdomain entries in the dataset contained pairs of two different PFAM domains. Reference structures had resolution ≤ 2.0 Å except for five of them (1BU1 and 1A19 taken from the Eyal et al dataset and 2HB2, 1WMG, 1ZWW taken into account to enrich the dataset with bigger domains and highly represented families).

### Source and analysis of atomic data on protein structures

An in-house relational database of protein structures (XMLRPDB) and the SCOWLP database [25,27] were used to obtain interaction information including solvent from X-ray structures in the PDB.

### Contact definition

Residue contacts in a reference structure were defined by following the physico-chemical criteria from SCOWLP [27]. We considered a 3.2 Å donor-acceptor distance for hydrogen bonds, 4 Å for salt bridges, and van der Waals radii for van der Waals interactions.

### Similarity matrices

We used the McLachlan similarity matrix (based on structural and genetic similarities of amino acids) as a "dry" matrix (DRY) [24]. To build a "wet" matrix (WET) we extracted information on protein interfacial residues and solvent from all available X-ray PDB structures using the SCOWLP database [25,27]. In this database, three classes of interacting residues are defined based on their interactions: dry (direct interaction), dual (direct and water-mediated interactions), and wet spots (residues interacting only through one water molecule). For each type of amino acid residue the probability of participation in water-mediated interactions (by establishing hydrogen bond by main chain or side chain) in protein interfaces was calculated as:

*p*_*i *_= *N*_*i*, *w*_/*N*_*i*, *total *_(Figure 1), where *i *corresponds to any of the 20 amino acids; *N*_*i*, *w *_is the number of the residues of this type forming wet spots or dual interactions; and *N*_*i*, *total*_is the total number of residues of this type participating in interfaces in all PDB structures. Each element of the WET similarity matrix was then defined as:

*WET*_*ij *_= *1*-|*p*_*i*_-*p*_*j*_|, where *i *and *j *correspond to any of the 20 amino acids.

The fact that for the creation of the wet matrix we take low resolution structures containing either none or few water molecules into account when considering the whole PDB does not bias the WET matrix because it affects each probability proportionally.

### Correlation coefficient calculations

For both DRY and WET similarity matrices the corresponding covariance matrices were calculated as previously described (Göbel et al 1994) using the formula:

, where *N *is the number of sequences; *i *and *j *are sequence position numbers; *S*_*ikl *_is a value from the similarity matrix (DRY or WET); *S*_*i *_is the mean of *S*_*ikl*_; σ_i _is the standard deviation of *S*_*ikl*_; and *W*_*kl *_is a weight matrix defined as:

, where *L *is the sequence length; *R*_*ik *_and *R*_*il *_are the residue types at position *i *in the sequences *k *and *l*, respectively; and δ is Kronecker delta [31].

For the interdomain dataset the weight matrix *W*_*kl *_was calculated as an average for the domains and weighted by sequence length. The positions with more than 10% of gaps as well as completely conserved positions were not included in the calculations (zero was assigned to the corresponding correlation coefficient). After calculating covariance matrices based on the DRY and WET similarity matrices, we built their linear combinations:

*r*_*ij *_= *r*_*ij DRY *_+ *α*·*r*_*ij WET*_, where *α *takes values from {0, 0.1, 0.2, 0.5, 1, 2, 4, 10, 20}, so that the weight ratio between the impact of DRY and WET represents the range from completely dry (*α = 0*) to extremely WET-biased covariance (*α = 20*).

### Evaluation of intradomain predictions

For evaluation of intradomain contacts predictions we used previously described methodology [4]. Sequence separation of 0, 6, 12 and 24 was used. Prediction *accuracy *was defined as the ratio between the number of correctly predicted contacts (C_corr_) and total number of predicted contacts (C_tot_). *Random accuracy *corresponds to the probability of correct prediction of the contact by chance and is equal to the ratio between experimentally observed contacts (C_obs_) and maximum number of possible contacts. The ratio between accuracy and random accuracy was introduced as *improvement ratio over random prediction*. *Wet prediction ratio *is equal to accuracy normalized by the accuracy obtained by using only the DRY matrix (α = 0). For the reference structures C_corr _was taken as the number of contacts defined by SCOWLP criteria (see the Contact definition section in Methods).

### Distance calculation and harmonic average (X_d_)

In the analysis of interdomain contacts the accuracy calculated in the same way as for intradomain contacts (typical value C_obs_~10^2^) is expected to be at least one order of magnitude lower (typical value C_obs_~10^1^). That is why comparison of accuracy, improvement ratio over random prediction and C_corr _as functions of *α *is not appropriate in this case. It has been shown that the distribution of distances between the correlated pairs is shifted to lower values compared to the distribution of distances for all residue pairs in two domains [9]. In our study we use a harmonic weighted difference statistic *X*_*d *_described before [9]:

, where n is the number of distance bins; *d*_*i *_is the upper limit for each bin normalized to the maximum value of the distributed distances; *P*_*ic *_is the percentage of the analyzed correlated pairs at the distances between *d*_*i *_and *d*_*i*-1_; and *P*_*ia *_is the same percentage for all pairs of residues. The width of bin was 4 Å. The higher the *X*_*d *_value, the more successful a prediction is.

Different definitions for the distance between residues resulted in all cases in the same trends and quantitatively only slightly affected *X*_*d *_values. For interdomain pairs we used distances between the centers of mass of residues in order not to be biased to either main-chain or side-chain contacts.

For *X*_*d *_calculations we took the best L/2 contacts for intradomain and (L_1_+L_2_)/2 contacts for interdomain contact predictions, where L_1 _and L_2 _are the reference sequences of the two interacting domains.

Although both the wet prediction ratio and *X*_*d *_characterize the predictive power of the method, it is irrelevant to compare the results obtained for these parameters with each other. The same applies to α values corresponding to best predictions.

### Statistical analysis

Statistical analysis of data was carried out with the R-package [32].

## Authors' contributions

SAS developed and implemented the WET similarity matrix and performed all the analysis. JT obtained the data from SCOWLP used for this work. GA obtained the data from XMLRPDB used for this work. SAS and MTP wrote the manuscript. MTP designed and supervised the project. All authors have read and approved the final manuscript.

## Supplementary Material

Additional file 1 **Probabilities for residues to be in contact with water in protein interfaces**. Probabilities for residues to be in contact with water in protein interfaces. The probabilities are derived from SCOWLP data for protein interfaces. Click here for file

Additional file 2 **Hydrophilicity index *vs *correlation for the DRY and WET matrices per residue type**. The grey shading highlights two areas resulting from the different trends. Click here for file

Additional file 3 **Dependence on *α *of wet prediction ratio for the intradomain dataset with sequence separation**. Sequence separation: A) 6. B) 12. C) 24. Click here for file
