# Supplementary material for: Analysis of the impact of solvent on contacts prediction in proteins
Source: BMC Struct Biol. 2009 Apr 15;9:22. doi: 10.1186/1472-6807-9-22 (PMC2676287; doi:10.1186/1472-6807-9-22)
Supplement: Additional file 1 — Probabilities for residues to be in contact with water in protein interfaces. Probabilities for residues to be in contact with water in protein interfaces. The probabilities are derived from SCOWLP data for protein interfaces. [file 1472-6807-9-22-S1.doc]

**Additional file 1. Probabilities for residues to be in contact with water in protein interfaces.**

| Residue | Total in interfaces | In contact with water | Probability |
| --- | --- | --- | --- |
| Gly | 131875 | 40188 | 0.30 |
| Ala | 133562 | 33008 | 0.25 |
| Val | 128573 | 21609 | 0.17 |
| Leu | 188008 | 29506 | 0.16 |
| Ile | 111915 | 18277 | 0.16 |
| Ser | 119168 | 50556 | 0.42 |
| Thr | 123482 | 47469 | 0.38 |
| Tyr | 114596 | 45580 | 0.40 |
| Phe | 106920 | 15936 | 0.15 |
| Trp | 42958 | 10448 | 0.24 |
| Pro | 104724 | 19398 | 0.19 |
| His | 71046 | 28339 | 0.40 |
| Met | 56221 | 8871 | 0.16 |
| Cys | 20393 | 4913 | 0.24 |
| Asp | 134113 | 78111 | 0.58 |
| Asn | 102592 | 55597 | 0.54 |
| Glu | 147932 | 77461 | 0.52 |
| Gln | 94758 | 46319 | 0.49 |
| Arg | 163652 | 86656 | 0.53 |
| Lys | 116322 | 47565 | 0.41 |

The probabilities are derived from SCOWLP data for protein interfaces.
