# Supplementary figures and images for: Analysis of the impact of solvent on contacts prediction in proteins
Source: BMC Struct Biol. 2009 Apr 15;9:22. doi: 10.1186/1472-6807-9-22 (PMC2676287; doi:10.1186/1472-6807-9-22)

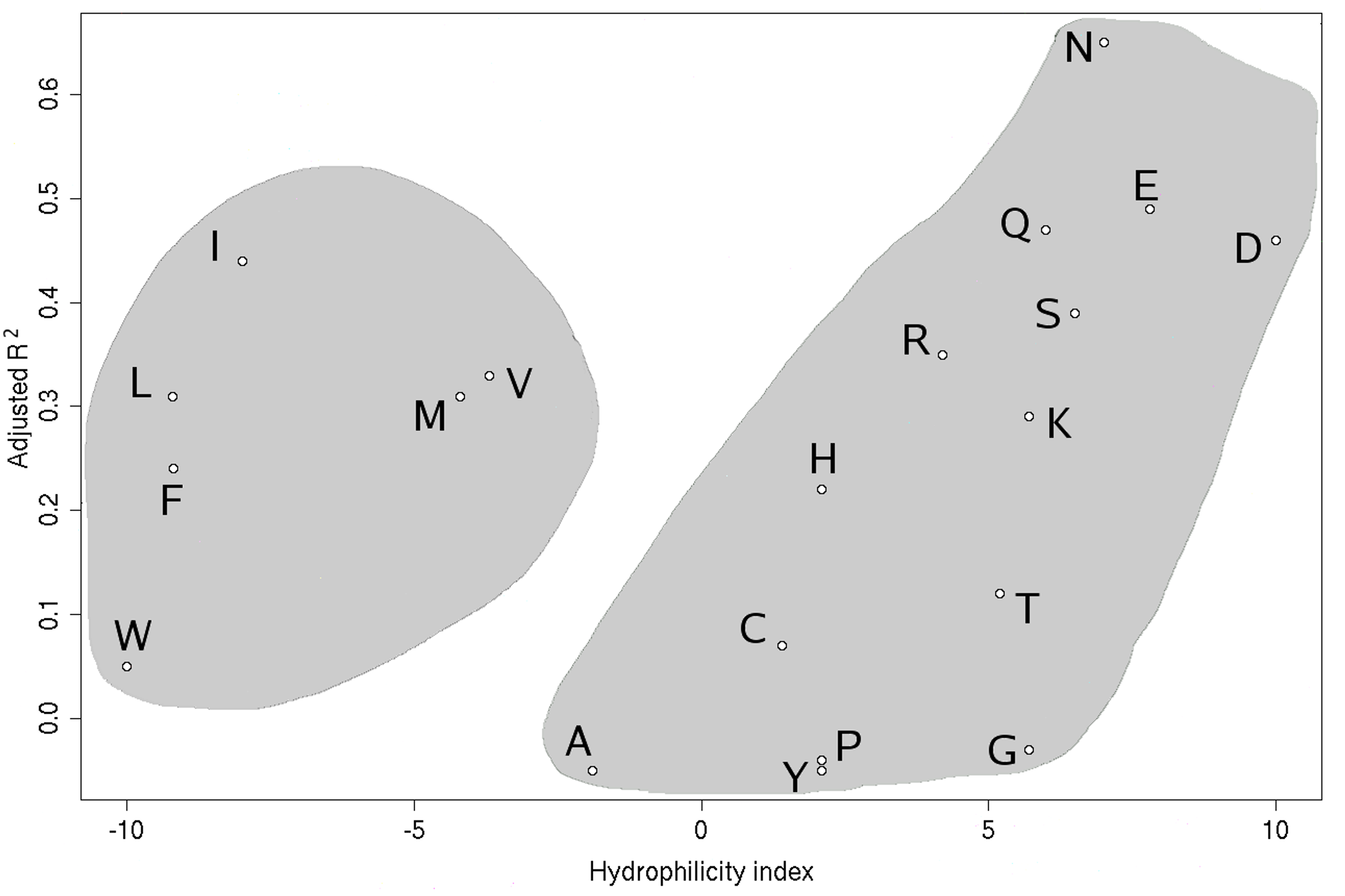

Supplement: Additional file 2 — Hydrophilicity index vs correlation for the DRY and WET matrices per residue type. The grey shading highlights two areas resulting from the different trends. [file 1472-6807-9-22-S2.tiff]

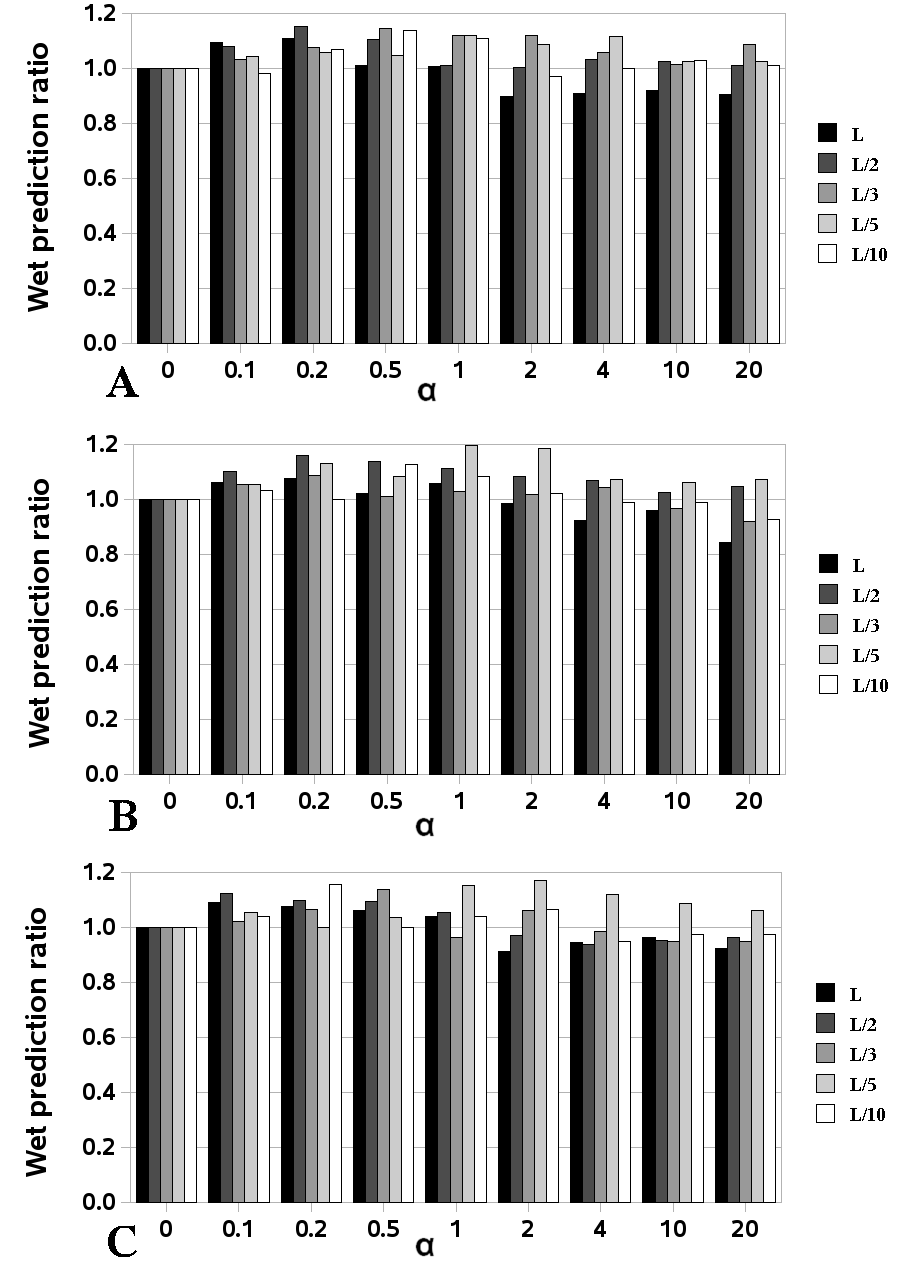

Supplement: Additional file 3 — Dependence on α of wet prediction ratio for the intradomain dataset with sequence separation. Sequence separation: A) 6. B) 12. C) 24. [file 1472-6807-9-22-S3.tiff]
